# Clinical Predictors of Prolonged Hospital Stay in Patients with Myasthenia Gravis: A Study Using Machine Learning Algorithms

**DOI:** 10.3390/jcm10194393

**Published:** 2021-09-26

**Authors:** Che-Cheng Chang, Jiann-Horng Yeh, Yen-Ming Chen, Mao-Jhen Jhou, Chi-Jie Lu

**Affiliations:** 1 Department of Neurology, Fu Jen Catholic University Hospital, Fu Jen Catholic University, New Taipei City 24352, Taiwan; changcc75@gmail.com (C.-C.C.); newtoloet@gmail.com (Y.-M.C.); 2 Ph.D. Program in Nutrition and Food Sciences, Human Ecology College, Fu Jen Catholic University, New Taipei City 242062, Taiwan; 3 School of Medicine, Fu Jen Catholic University, New Taipei City 242062, Taiwan; M001074@ms.skh.org.tw; 4 Department of Neurology, Shin Kong Wu Ho-Su Memorial Hospital, Taipei 11101, Taiwan; 5 Department of Neurology, Kaohsiung Medical University, Kaohsiung 80708, Taiwan; 6 Graduate Institute of Business Administration, Fu Jen Catholic University, New Taipei City 242062, Taiwan; aaa73160@gmail.com; 7 Artificial Intelligence Development Center, Fu Jen Catholic University, New Taipei City 242062, Taiwan; 8 Department of Information Management, Fu Jen Catholic University, New Taipei City 242062, Taiwan

**Keywords:** myasthenia gravis, risk factors, corticosteroids, machine learning

## Abstract

Myasthenia gravis (MG) is an autoimmune disorder that causes muscle weakness. Although the management is well established, some patients are refractory and require prolonged hospitalization. Our study is aimed to identify the important factors that predict the duration of hospitalization in patients with MG by using machine learning methods. A total of 21 factors were chosen for machine learning analyses. We retrospectively reviewed the data of patients with MG who were admitted to hospital. Five machine learning methods, including stochastic gradient boosting (SGB), least absolute shrinkage and selection operator (Lasso), ridge regression (Ridge), eXtreme gradient boosting (XGboost), and gradient boosting with categorical features support (Catboost), were used to construct models for identify the important factors affecting the duration of hospital stay. A total of 232 data points of 204 hospitalized MG patients admitted were enrolled into the study. The MGFA classification, treatment of high-dose intravenous corticosteroid, age at admission, treatment with intravenous immunoglobulins, and thymoma were the top five significant variables affecting prolonged hospitalization. Our findings from machine learning will provide physicians with information to evaluate the potential risk of MG patients having prolonged hospital stay. The use of high-dose corticosteroids is associated with prolonged hospital stay and to be used cautiously in MG patients.

## 1. Introduction

Myasthenia gravis (MG) is an autoimmune disorder that affects the postsynaptic muscle membrane of the neuromuscular junction (NMJ) and can cause skeletal muscle weakness [1]. Its prevalence in Taiwan is approximately 84 to 140 per million persons [2]. It presents in varying degrees and with a combination of weakness in the ocular, bulbar, limb, and respiratory muscles in fluctuation. The pathogenesis includes antibody-mediated immunologic attack of the receptors in the postsynaptic membrane, directed against acetylcholine receptors (AchR), muscle-specific kinase (MuSK), and lipoprotein-related protein 4 (LRP4) [3,4]. In the majority of MG patients, the thymus undergoes structural and functional changes that are characterized by the development of a thymoma or follicular hyperplasia [1]. Ten percent of patients with AChR-MG had thymomas, whereas some patients had thymic hyperplasia, and the prevalence increased with age [1].

The treatment of MG includes symptomatic therapy, such as anticholinergic medications, immunosuppressants, and thymectomy [5]. Immunosuppressants include corticosteroids, azathioprine, and various other medications. Guidelines also recommend thymectomy for patients with thymomas or early-onset MG with thymic hyperplasia [6,7]. Once an acute exacerbation of MG occurs, short-acting immunomodulating treatments such as plasmapheresis and intravenous immunoglobulin (IVIG) should be administered [7]. In some centers, such as our center, some of the patients with MG exacerbation are treated with high doses of intravenous corticosteroids. However, approximately 38% of MG patients experience remission, and 10% of them are refractory to the conventional rescue therapy that requires prolonged hospitalization, which is the major cause of morbidity [8]. Many studies have evaluated the predictors of prognosis in patients with MG. However, less attention has been paid to the significance of the factors associated with treatment outcomes in hospitalized MG patients. Our study aimed to identify the important factors that predict the prognosis and duration of hospitalization in patients with MG by using machine learning methods. A total of 21 factors associated with the clinical condition were chosen for machine learning analyses. Five machine learning techniques were used to identify the factors that predict the duration of hospitalization. Our goal was to aid physicians to identify factors that predict prolonged hospital stay in patients with MG by providing analytical data for clinical consideration.

## 2. Methods

This study applied five machine learning methods, namely, stochastic gradient boosting (SGB), least absolute shrinkage and selection operator (Lasso), ridge regression (Ridge), eXtreme gradient boosting (XGboost), and gradient boosting with categorical features support (Catboost), to construct predictive models for predicting hospital staying in patient with MG and to evaluate the importance of different treatments and factors for MG affecting the inpatient days. The machine learning algorithms have been widely used in different neurology areas [9,10,11,12].

For the first method, SGB is a hybrid tree algorithm that combines boosting and bagging techniques and uses the gradient descent technique to minimize the loss function [13,14]. In the SGB algorithm, trees are grown sequentially and each tree is a weak learner that is grown using information from previously grown trees. With each tree modeling the errors, the newly added decision tree fits the residuals from the current decision tree [15].

Lasso, the second method of this study, is a developed shrinkage regularization algorithm in the linear model [16]. The Lasso principle is to the sum absolute values of the coefficients and minimizing the sum of squared residuals. It uses the penalty parameter to control the trade-off between the bias and parsimony of a fitted Lasso model. The Lasso performs via a continuous coefficient shrinking operation, for a large reduction in the variance of the predicted values that minimize regression coefficients in order to reduce the likelihood of overfitting [17,18].

In the third method, Ridge is an improved least squares estimation method. The Ridge is based on the same principles as the Lasso method—that is, the shrinkage regularization method. The Ridge principle is to use a sum-of-squares error function and regularization technique to control the trade-off between bias and variance [19]. In the regularization process of Ridge, it adds appropriate penalties to the model that shrinks all the coefficients to a nonzero value or approach zero, and then minimizes sum squared error to avoid large coefficients and, thus, help reduce overfitting [20].

XGboost, the fourth method of this study, is a tree-based gradient boosting learning algorithm that uses ensemble learning technology [21]. Its principle is to achieve accurate classification by iteratively training many weak classifier models. The Taylor binomial expansion is used in XGBoost to approximate the objective function and arbitrary differentiable loss functions [22]. XGboost applies a new distributed algorithm to accelerate tree building process and alleviate the overfitting problem [23,24].

For the fifth method, Catboost is a novel decision tree algorithm that combines gradient boosting and classification features based on the input feature of the ordered boosting method [25]. Its principle is to use an iterative approach, based on decision trees as weak base learners, to generates many tree models. In an iterative process, it integrates all combinations and classification features of the current tree into a sequence to generate random multiple permutations and a final model. Catboost can reduce the deviation of the gradient estimation and improve the generalization ability by utilizing ordered boosting of the gradient estimation method in the algorithm [25,26].

In this study, all methods were implemented in R software version 3.6.2 (R core team, Vienna, Austria) [27] and RStudio of version 1.1.453 [28]. The algorithms for the methods are based on the relevant R packages. For the SGB method, the “gbm” R package of version 2.1.8 was used [29]. XGboost was implemented by the “XGboost” R package of version 1.4.1.1 [30]. Catboost was computed by the “catboost” R package of version 0.25.1 [31]. To estimate the best parameter set for developing effective SGB, XGboost, and Catboost models, the “caret” R package version 6.0-84 was used for tuning the relevant hyperparameters [32]. Lasso and Ridge were implemented by the “glmnet” R package of version 4.1-1; the default setting was used to construct the models [33].

Approximately 80% of the total data was randomly selected as the training data set, and the remaining 20% was treated as the out-of-sample testing data set. This study used the 10-fold cross-validation method to estimate the best hyperparameters of each model since it could acquire a relatively stable evaluation of the methods [34]. This study applied accuracy, sensitivity, specificity, and AUC (area under the receiver operating characteristic (ROC) curve) values as performance metrics to evaluate the performance of the five machine methods [35].

## 3. Empirical Study

### 3.1. Dataset

**Figure 1:**
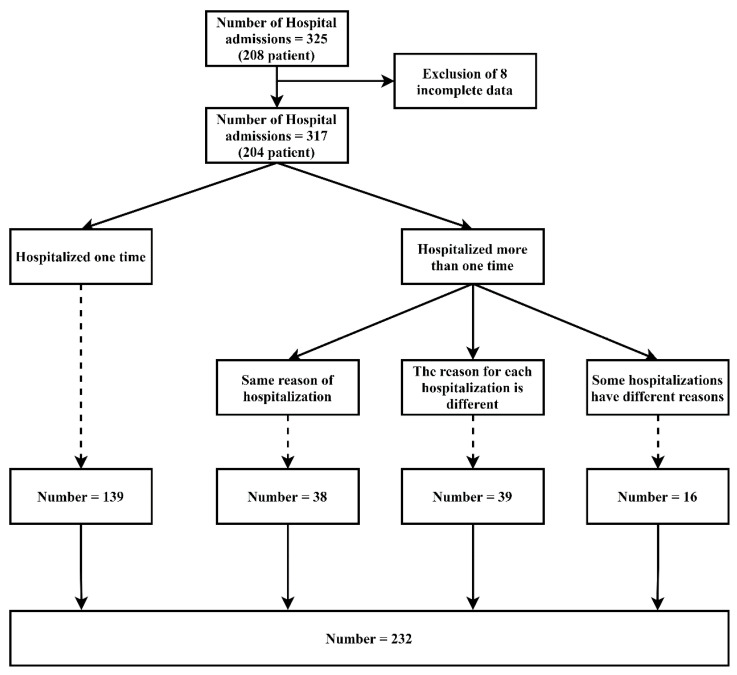
Algorithm of case identification.

We retrospectively reviewed the data of 513 hospital admissions of patients with MG who were admitted to the Shin-Kong Wu Ho-Su Memorial Hospital in Taipei, Taiwan, between December 2015 and October 2018. We excluded 188 hospital admissions because they were not due to MG and eight others because of data loss. After cleaning, the data from 317 hospital admissions data points of 204 patients were used for the analyses. We merged the data in case the same patient had been hospitalized for the same reason. Finally, 232 data points were used for the analysis (Figure 1). Concerning ethical issues with regard to the use of the dataset, the protocol of this study was evaluated and deemed acceptable by the Research Ethics Review Committee of the Shin Kong Wu Ho-Su Memorial Hospital (No. 20190109R).

We retrospectively reviewed medical records, including age, sex, age at diagnosis, disease duration, reason for hospitalization, disease severity, autoantibody serology status, medications, maximum dosage of corticosteroid before admission, thymic histology, history of thymectomy, treatment during hospitalization, and length of hospital stay. Disease severity was graded according to the classification of the Myasthenia Gravis Foundation of America (MGFA) classification at admission. A total of 21 factors associated with prolonged hospital stay were collected. The reasons for hospitalization were divided into five categories: admission for thymectomy, acute exacerbation of MG symptoms, pneumonia, influenza infection, and admission for intravenous rituximab administration. The MGFA clinical classification was based on previous reviews that represented the patient’s clinical severity upon admission (Day 0). The maximum daily oral steroid dose before admission was recorded from the dosages during outpatient visits one month before admission.

History of thymectomy was divided into three categories: (1) Patient has never undergone thymectomy. (2) Underwent thymectomy during this admission. (3) Thymectomy had been performed previously. The treatment during hospitalization included plasmapheresis; intravenous IVIG; rituximab; and, in our center, some of the patients with MG exacerbation are treated with a high dose of intravenous corticosteroids. Treatment with plasmapheresis was divided into three categories: (1) Patient did not undergo plasmapheresis. (2) Patient underwent less than five sessions. (3) Patient underwent more than 5 sessions. Plasmapheresis is the standard first-line treatment of our hospital for worsening of MG symptoms and before elective thymectomy.

The serology status of MG autoantibodies included anti-AChR-antibody and anti-MuSK-antibody positivity or negativity as well as double seronegativity. Finally, the duration of hospital stay was divided into two categories: hospital stay of more than 14 days and less than 14 days.

### 3.2. Results

The 21 variables considered as impact factors for prolonged hospitalization (Y) in patients with MG are shown in Table 1. There were five categories of reasons for admission (V5): 60 (25.86%) patients were admitted for thymectomy, 125 (53.88%) patients due to acute exacerbation of MG symptoms, 39 (16.81%) patients due to pneumonia, 5 (2.16%) due to influenza infection, and 3 for rituximab injection to control MG symptoms. The gender (V4) distribution was 61.64% females, and the average age at admission (V1) was 49.43 ± 17.14 years. The disease duration (V2), defined as the time from the onset to the first visit after 1 December 2015, was 67.56 ± 84.14 months. The average age at onset (V3) of MG symptoms was 42.47 ± 18.18 years. Five patients died during admission (5/208, 2.5%). The average duration of hospital stay (Y) was 13.93 days.

All patients were classified into eight groups according to the MGFA clinical classification at admission (V6): class I (ocular type), 24 patients (10.34%); class II, 91 patients (39.23%); class III, 74 patients (31.9%); class IV, 27 patients (11.64%); MG crisis, 16 patients (6.9%). Regarding the medications used, 141 patients (60.78%) were treated with different oral immunosuppressants, including azathioprine, mycophenolate, and tacrolimus (V14–V17). The maximum dose of prednisolone was 14.35 ± 15.63 mg/day.

Thymus histology was as follows: 110 patients (47.41%) had thymoma and 67 patients (67%) had thymic hyperplasia. A total of 148 patients (63.8%) had undergone thymectomy before or after admission. After hospitalization, 162 (69.83%) patients underwent plasmapheresis, 15 (6.47%) patients received intravenous immunoglobins, 43 (18.53%) patients received intravenous corticosteroids, and 6 (2.59%) patients received rituximab.

The combination of immunomodulation therapy is shown in Table 1. For the serology status of the autoantibodies, anti-AChR-antibody positivity was 87.93%, anti-MuSK-antibody positivity was 4.74%, and double seronegative status was 7.76%.

**Figure 2:**
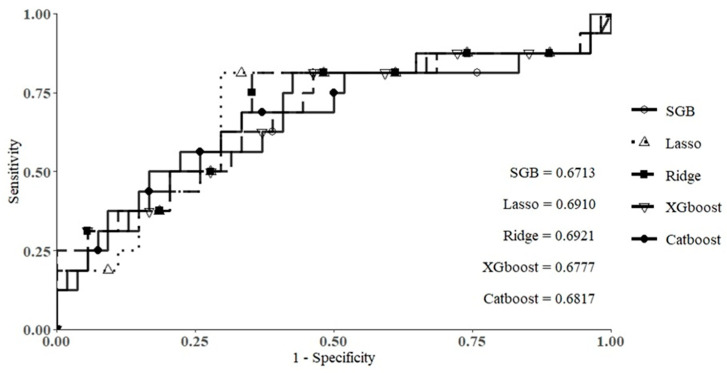
ROC curves of the five methods.

This study used SGB, Lasso, Ridge, XGboost, and Catboost methods to construct predictive models for hospital stay in patients with MG. Table 2 shows the prediction performance of the five models. As shown in the table, the AUC values of the SGB, Lasso, Ridge, XGboost, and Catboost models were 0.6713, 0.6910, 0.6921, 0.6777, and 0.6817, respectively. The Ridge model provided the highest AUC value, followed by the Lasso, Catboost, XGboost, and SGB models. Figure 2 shows the ROC curves of the five machine learning methods. This figure also shows that the Ridge method showed the best predictive ability compared with the other four methods. From Table 2 and Figure 2, it can be seen that the Ridge method can generate better performance than the other four methods as it generates the best AUC and specificity values and provides relatively higher accuracy and sensitivity values. Therefore, Ridge was the best predictive model in this study for predicting the duration of hospital stay in patients with MG.

In order to assess the important factors affecting the duration of hospital stay in patients with MG, ranking of the importance of each variable within different models can provide useful information because the five machine learning methods generated similar prediction performance. To generate the ranking value of each factor, the “caret” R package of version 6.0-84 [32], based on the embedded method, was implemented for each of the five methods. The factor with the largest importance value was ranked number one. Conversely, the factor with the lowest or zero importance value was ranked last.

**Figure 3:**
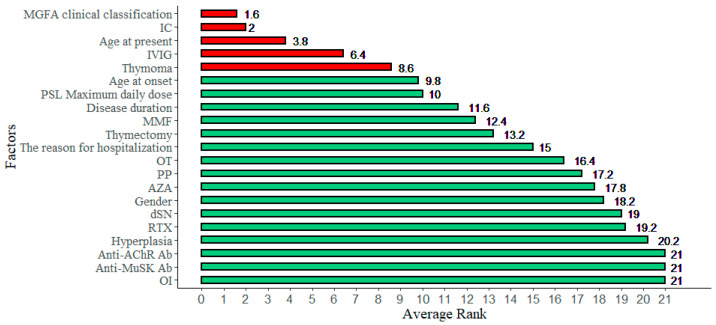
The averaged ranked importance values of factors.

Table 3 illustrates the importance ranking of each factor generated by the SGB, Lasso, Ridge, XGboost, and Catboost methods. In the table, it can be seen that different methods generated different relative importance ranks for each factor. For example, in the Ridge method, the first three important factors are V18, V6, and V20. However, in the Lasso method, the most important factor is V6, followed by V18 and V20. To fully consider the importance of each factor in all five methods, an average ranking was obtained by averaging the rank value of each variable in each method. Figure 3 depicts the factors based on the increasing order of average ranking values. Following physicians’ suggestions, the top five important variables—V6 (MGFA clinical classification), V20 (IC), V1 (Age at admission), V18 (IVIG), and V7 (Thymoma)—were selected as the crucial factors that affect the duration of hospital stay in patients with MG.

## 4. Discussion

To the best of our knowledge, no previous study has used machine learning to identify the factors that can predict the outcomes of MG. The majority of admitted MG patients in this study were females (71.6% vs. 28.4%), which corresponds to the distribution of the disease [36]. The major reason for admission was acute exacerbation of symptoms. The MGFA clinical classification before admission (V6), treatment with intravenous corticosteroid (V20), age at admission (V1), treatment with intravenous immunoglobulins (IVIG) (V18), and presentation of thymoma (V7) were the top five significant variables for evaluating the effect on prolonged hospitalization of patients with MG.

Corticosteroids were the most commonly used immunosuppressant medications for MG. Our study showed that treatment with high-dose intravenous corticosteroid (V20) was an accurate predictive factor for prolonged hospital stay in patients with MG. In recent years, immunosuppressant therapies have been well-established and have become a major management strategy for patients with MG. Corticosteroid treatment is an effective first-line immunosuppressive therapy in MG because it can decrease leukocyte adhesion to the epithelial membrane and decrease inflammatory cytokines [37]. Oral corticosteroids are generally used when the symptoms of MG are not adequately controlled by cholinesterase inhibitors alone and are suitable for diseases causing milder disability; high-dose intravenous corticosteroid pulse therapy has been reported to produce rapid improvement in moderate-to-severe MG [38]. The early fast-acting treatment strategies in Japan used in MG treatment during hospital admission include plasmapheresis followed by the administration of high-dose intravenous corticosteroids [39,40]. However, intravenous corticosteroids have numerous side effects, including weight gain, diabetes, and hypertension, and they can also paradoxically worsen MG symptoms if given in high doses, especially in the first 7–10 days after starting treatment, which can last for several days [41]. One study has also shown decreased muscle strength after intravenous corticosteroid infusion [38]. The mechanism of steroid-induced exacerbation is not yet well-established; antibodies released by degraded lymphocytes may increase cholinesterase activity at the neuromuscular junction and increase the immune response [42,43,44]. It is possible that the transient worsening of MG symptoms may cause prolonged hospitalization, and physicians need to pay attention to this.

Intravenous immunoglobulin (IVIG) is the second-line immunomodulation therapy and has been demonstrated to be safe and efficient for the treatment of MG. Several clinical trials have shown that IVIG is as effective as plasmapheresis for controlling acute exacerbations of MG [45,46]. The mechanism of action of IVIG involves the inhibition of inflammatory cytokines and complement deposition [47]. The international consensus guidelines recommend that plasmapheresis and IVIG be appropriately used in patients with life-threatening MG, such as those with respiratory insufficiency [48]. Although they may have the same effectiveness, reports suggested that plasmapheresis is recommended as the first-line therapy due to its more rapid effect [49]. Therefore, IVIG was administered to patients when they deteriorated or had a poor response after first-line therapy (plasmapheresis and/or intravenous corticosteroid), which is why treatment with IVIG (V18) was another significant factor that influenced the duration of hospital stay. In a previous retrospective study, contrary to our findings, a similar duration of hospital stay was found in the group that received IVIG and the one that did not. This could be explained by the different considerations for treatment with IVIG; its use depends on the presence of respiratory distress, medical comorbidities, access to it, and its cost in different countries [50].

The MGFA clinical classification before admission (V6) and presentation of thymoma (V13) were the other significant factors predicting prolonged hospital stay. The MGFA clinical classification is used to identify the different clinical features and severity of patients with MG. It divides MG into five main classes and several subclasses [51]. The MGFA clinical class before admission (V6) reflects the severity of MG, which indicates that the severity of the symptoms may play a critical role in prolonged hospitalization.

Forty seven percent of the MG patients in this cohort had a thymoma. Surgery is the mainstay modality for the management of thymoma. Some studies have reported a worse prognosis in thymomas with MG [52,53]. The MG symptom relief rate following surgery has been found to be 70–80%. Zhang et al. demonstrated that the survival rate of thymoma patients with MG (76.0%) was lower than that of patients without MG (89.1%, *p* = 0.026) [52]. However, a previous study revealed that the prognosis was similar between patients with and those without MG [54]. Further studies should focus on the types of thymomas to evaluate their influence on hospital stay.

The age at admission (V1) also had a significant influence on hospital stay. One retrospective study showed that late-onset MG, defined as onset age > 65 years, may be more prone to increased disease severity [55]. In contrast to previous reviews, our study demonstrated that the age at admission (V1) is more important than the age at onset (V3). Older age may indicate a longer duration of the disease course and more fragility to disease progression. Elderly patients with MG tend to have more complications and comorbidities and may result in longer hospital stay. Due to the retrospective nature of this study, we could not analyze which comorbidities contributed to increased length of hospital stay in the elderly MG patients.

The basic principle of machine learning (ML) is its predictive performance on unseen data that assists doctors in improving the care quality and making more precise decisions [56]; it can play a critical role for predicting the prognosis of MG. ML algorithms can automatically learn useful data representations and process different types of input data. Thus, ML fills a significant gap in learning from clinical experience that is capable of predicting outcomes, detecting features, and optimizing management [57]. Instead of a single set of multiplications, ML methods can leverage multiplications and other mathematical operations to extract descriptive features of complex input data and try to explore possible nonlinear relationships and higher-order interactions between the risk factors. However, the current knowledge about the reliability of ML in MG is limited. In our study, the treatment with IVIG and intravenous corticosteroid had been two factors that influence the hospital staying. We use five ML methods with different characteristics to reduce selection bias in the treatment of IVIG or IC. ML in our studies, in contrast to statistical modeling, examines possible nonlinear relationships and higher-order interactions of input data to generate a prediction model that maximizes predictability. Our results showed that integrating the feature selection results of the five ML methods can provide useful ranking information about risk factors for clinical practice a promising predictive accuracy. For further studies, the decision-tree-based machine learning algorithms can be used to help us explore inner structure of nonlinear and higher-order interactions of risk factors.

Previous studies have discussed the predictors of MG deterioration, severity, and hospital stay. Suzuki et al. reported that patients with anti-Kv1.4 antibodies had more prominent MG progression than those without them [58]. Wakata et al. found that thymic hyperplasia occurred more commonly in relapsed cases [8]. Lili Wang et al. showed that the co-occurrence of other autoimmune diseases can serve as a potential predictor of symptom deterioration [59]. A cross-sectional study in Thailand showed that pneumonia may result in a higher mean duration of hospital stay and poorer outcomes [50]. In our study, unlike previous studies, the proportion of hospitalizations due to pneumonia was 16.8%, but pneumonia did not significantly affect the duration of hospital stay of patients with MG. We also found that the mortality rate during the hospital course was 2.5%, which is similar to reports from the US [36].

There are some limitations to our study: First, since this was a retrospective study, some of the specific details such as the quantitative myasthenia gravis score were not available for analysis. Second, the samples were collected from a single medical center. Third, these variables were chosen based on clinical data, and other variables such as total cumulative dose of corticosteroid, total dose of immunosuppressant, period of treatment, and the comorbidities, were not included in our analysis. Finally, we analyzed the MGFA classification at the patient admission to hospital; however, we did not assess MGFA classification in a fixed time frame before admission or after treatment.

## 5. Conclusions

This is the first study that uses a machine learning approach to develop a model to predict the hospital staying in patients with MG. We demonstrate, based on the integrated information of five ML methods, that the MGFA clinical classification before admission, treatment with high-dose intravenous corticosteroid, age at admission, treatment with IVIG, and presence of thymoma significantly influenced the duration of hospital stay. Our data confirm the notion that use of high doses of corticosteroids may prolong the length of hospitalization in MG patients and has to be avoided if possible. In addition, thymoma related to MG needs close observation during the course of hospital stay. We hope that our ML models approach will shade more light on the duration of hospital stay in patients with MG and develop a clinical prediction supportive tool to further improve MG patient care.

## Figures and Tables

**Table 1:** Subject Demographics.

Characteristics	Metrics
Basic Information:	Mean ± SD
V1 Age at admission (year-old)	49.43 ± 17.14
V2 Disease duration (months)	67.56 ± 84.14
V3 Age at onset (year-old)	42.47 ± 18.18
V4 Gender:	*n* (%)
Male	89 (38.36%)
Female	143 (61.64%)
V5 The reason for hospitalization:	*n* (%)
1: Thymectomy	60 (25.86%)
2: Acute exacerbation of MG	125 (53.88%)
3: Pneumonia	39 (16.81%)
4: Influenza	5 (2.16%)
5: Hospitalization for Rituximab	3 (1.29%)
V6 MGFA clinical classification:	*n* (%)
1: Class I: ocular muscle weakness	24 (10.34%)
2: Class IIA: Mild limbs, axial predominant weakness	27 (11.64%)
3: Class IIB: Mild bulbar and respiratory predominant weakness	64 (27.59%)
4: Class IIIA: Moderate limbs, axial predominant weakness	16 (6.90%)
5: Class IIIB: Moderate bulbar and respiratory predominant weakness	58 (25.00%)
6: Class IVA: Severe limbs, axial predominant weakness	NA
7: Class IVB: Severe bulbar and respiratory predominant weakness	27 (11.64%)
8: Class V: Intubation	16 (6.90%)
Thymus:	*n* (%)
V7 Thymoma:	
0: No	122 (52.59%)
1: Yes	110 (47.41%)
V8 Hyperplasia:	
0: No	165 (71.12%)
1: Yes	67 (28.88%)
V9 Thymectomy:	
0: No	84 (36.21%)
1: Underwent thymectomy during this admission	93 (40.09%)
2: Had undergone thymectomy before	55 (23.71%)
Autoantibody:	*n* (%)
V10 Anti-AChR Ab:	
0: No	28 (12.07%)
1: Yes	204 (87.93%)
V11 Anti-MuSK Ab:	
0: No	221 (95.26%)
1: Yes	11 (4.74%)
V12 dSN:	
0: No	214 (92.24%)
1: Yes	18 (7.76%)
Treatment status:	Mean ± SD
V13 PSL Maximum daily dose (mg)	14.35 ± 15.63
V14 OI:	*n* (%)
0: No	91 (39.22%)
1: Yes	141 (60.78%)
V15 AZA:	*n* (%)
0: No	156 (67.24%)
1: Yes	76 (32.76%)
V16 MMF:	*n* (%)
0: No	223 (96.12%)
1: Yes	9 (3.88%)
V17 OT:	*n* (%)
0: No	226 (97.41%)
1: Yes	6 (2.59%)
V18 IVIG:	*n* (%)
0: No	217 (93.53%)
1: Yes	15 (6.47%)
V19 PP:	*n* (%)
0: No	70 (30.17%)
1: 5 sessions	131 (56.47%)
2: >5 sessions	31 (13.36%)
V20 IC:	*n* (%)
0: No	189 (81.47%)
1: Yes	43 (18.53%)
V21 RTX:	*n* (%)
0: No	226 (97.41%)
1: Yes	6 (2.59%)
Y Hospital stay timing:	*n* (%)
0: Less than 14 days hospital stay	176 (75.86%)
1: More than 14 days hospital stay	56 (24.14%)

Note: Anti-AChR Ab—anti-acetylcholine receptor; Anti-MuSK Ab—muscle-specific receptor tyrosine kinase; dSN—Double-seronegative; PSL—prednisolone; OI—Oral Immunosuppressant; AZA—Azathioprine; MMF—Mycophenolate mofetil; IVIG—Intravenous immunoglobins; PP—Plasmapheresis; IC—Intravenous corticosteroid; RTX—Rituximab; OT—Oral Tacrolimus.

**Table 2:** The performance of the SGB, Lasso, Ridge, XGboost, and Catboost methods.

Methods	Accuracy	Sensitivity	Specificity	AUC
SGB	0.6286	0.5741	0.8125	0.6713
Lasso	0.7286	0.7037	0.8125	0.6910
Ridge	0.6857	0.6482	0.8125	0.6921
XGboost	0.6000	0.5370	0.8125	0.6777
Catboost	0.6714	0.6667	0.6875	0.6817

Note: SGB—stochastic gradient boosting; Lasso—least absolute shrinkage and selection operator; Ridge—ridge regression; XGboost—eXtreme gradient boosting; Catboost—gradient boosting with categorical features support.

**Table 3:** The ranked importance of each factor using the SGB, Lasso, Ridge, XGboost, and Catboost methods.

Factors	SGB	Lasso	Ridge	XGboost	Catboost	Average Rank
V1: Age at admission	3	4	4	4	4	3.8
V2: Disease duration	5	21	21	5	6	11.6
V3: Age at onset	4	21	8	9	7	9.8
V4: Gender	21	21	21	10	18	18.2
V5: The reason for hospitalization	9	21	21	21	3	15
V6: MGFA clinical classification	2	1	2	2	1	1.6
V7: Thymoma	8	6	7	7	15	8.6
V8: Hyperplasia	21	21	21	21	17	20.2
V9: Thymectomy	7	21	21	8	9	13.2
V10: Anti-AChR Ab	21	21	21	21	21	21
V11: Anti-MuSK Ab	21	21	21	21	21	21
V12: dSN	21	21	21	21	11	19
V13: PSL Maximum daily dose	6	21	9	6	8	10
V14: OI	21	21	21	21	21	21
V15: AZA	10	21	21	21	16	17.8
V16: MMF	21	5	5	21	10	12.4
V17: OT	21	21	6	21	13	16.4
V18: IVIG	21	2	1	3	5	6.4
V19: PP	21	21	9	21	14	17.2
V20: IC	1	3	3	1	2	2
V21: RTX	21	21	21	21	12	19.2

Note: Anti-AChR Ab—anti-acetylcholine receptor; Anti-MuSK Ab—muscle-specific receptor tyrosine kinase; dSN—Double-seronegative; PSL—prednisolone; OI—Oral Immunosuppressant; AZA—Azathioprine; MMF—Mycophenolate mofetil; IVIG—Intravenous immunoglobins; PP—Plasmapheresis; IC—Intravenous corticosteroid; RTX—Rituximab; OT—Oral Tacrolimus.

## Data Availability

Data available on request due to privacy/ethical restrictions.
